# Design and Fabrication of Random Metal Foam Structures for Laser Powder Bed Fusion

**DOI:** 10.3390/ma12081301

**Published:** 2019-04-20

**Authors:** Nicola Contuzzi, Sabina Luisa Campanelli, Fabrizia Caiazzo, Vittorio Alfieri

**Affiliations:** 1 Dip. di Meccanica, Matematica e Management—Politecnico di Bari, Viale Japigia 182, 70126 Bari (BA), Italy; nicola.contuzzi@poliba.it (N.C.); sabinaluisa.campanelli@poliba.it (S.L.C.); 2 Dip. di Ingegneria Industriale—Università degli Studi di Salerno, Via Giovanni Paolo II 132, 84084 Fisciano (SA), Italy; valfieri@unisa.it

**Keywords:** additive manufacturing, design for manufacturing, random foam structures

## Abstract

With the development of additive manufacturing, the building of new categories of lightweight structures such as random foams have been offered. Nevertheless, given the complexity of the required parts, macroscopic defects may result or the process may even fail. Therefore, proper actions must be taken at the design stage. In this paper, a method of design for additive manufacturing (DfAM) to build metal random foam structures is proposed. Namely, a procedure is suggested to generate a structure that has interconnected porosity. This procedure is based on the aimed fractional density and several technical requirements, and then the geometry is optimized and meshed. To validate the algorithm, a test article consisting of a metal cylinder with spherical random pores ranging from 1 to 6 mm in diameter with a resulting fractional density of 40 ± 2% has been conceived and manufactured by means of laser powder bed fusion (LPBF). On the basis of the outcome of the manufacturing process, crucial information has been gathered to update the algorithm.

## 1. Introduction

Lightweight metal structures are widely used in aeronautics, automotive, biomedical [1], energy, and bionics [2] fields. Namely, high strength-to-weight ratio, thermal and acoustic insulation, good properties of energy absorption, and even electromagnetic shielding [3] benefit when metal cellular materials are considered [4].

For the purpose of designing and building, many different approaches have been discussed in the literature. Interestingly, the concept has been significantly addressed in the field of additive manufacturing (AM), as state-of-the-art and flexible processes have been developed that offer new opportunities in terms of shapes, sizes, geometric mesostructures, material compositions, and microstructures, and therefore improve both performance and life-cycle. Design for additive manufacturing (DfAM) has been introduced for the purpose of exploiting all the opportunities in AM [5,6]. By considering this, the production of freeform complex structures using potentially a wide range of materials, including high-performance metals, has been allowed [7,8].

Periodic structures are the most common cellular materials, given their capability to provide variations in the structural properties and benefit controllable deformation. With respect to this subject, several methods for designing lattice structures with controlled anisotropy are reported [9]. As a matter of fact, lattice-truss structures are affected by anisotropy resulting in weaker directions depending on the arrangement of the trusses [10]. Nevertheless, since the direction of loading is not known in advance of the specific applications (e.g., aerospace or medical), a lightweight structure is required to exhibit a similar homogeneous mechanical behavior, hence the foams, i.e., samples with random pore distribution [11], represent a valid alternative to lattice-truss structures. On the other hand, the conventional production of foams is currently limited to low-melting-point metals, such as aluminum or copper. Consequently, other materials with higher strength must be investigated, and therefore AM processes represent a valid alternative and are worth investigating for this purpose.

In general, foam samples are designed using the reconstruction methods of either statistical or stochastic, for example, a three-dimensional porous media is created from two-dimensional (2D) images or a three-dimensional (3D) cloud of data points [12]. In this context, scanning electron microscopy [13], computed tomography, and X-rays scanning have been considered [14] to generate the models. Then, different fabrication techniques can be compared. In the literature, the methods of bubbling gas or injecting a foaming agent in the molten alloy are referred to as conventional approaches [3], and in the latter, expanding hydrogen is released, and therefore used to create pores [15]. Reviews of other consolidated technologies such as the space-holder method [16] or the continuous zone melting technique [17] are available in the literature. When moving to more recent methods, AM is certainly suitable due to its advantages. For example, with specific reference to the building of metal random foam structures, the advantage of control over size, density, and local distribution of the designed porosity is documented [3]. In particular, layer-by-layer AM building (i.e., two-stage processing) is expected to provide higher accuracy as compared with directed deposition AM (i.e., one-stage processing) [18]. The feasibility of manufacturing similar-to-foam steel components with spherical porosity adopting laser powder bed fusion (LPBF) has been explored [4], but limited research has been devoted to the manufacturing of metal random foam structures, which have some issues that must be addressed. First, interconnected porosity is mandatory in order to allow the extraction of loose powder from cavities. Moreover, a proper size of pores is required to comply with the manufacturing capability of the building machine to prevent inner supporting structures [19], which otherwise should be removed in post-processing.

To address this lack of knowledge, porous structures with random spherical pores and controlled fractional density are designed and manufactured in this article. A DfAM approach is proposed and the main issues are discussed. Namely, a three-dimensional porous structure is conceived with given porosity, then the geometry is converted to optimized points and eventually meshed. Possible errors, in terms of shapes and poor contours, are corrected. Prior to manufacturing, the structure is checked layer-by-layer to assess its effective manufacturability. During manufacturing via LPBF, several post-processing checks are conducted, and then crucial findings are drawn to update the algorithm for random foam generation.

## 2. Materials and Methods 

### 2.1. Design of a Random Foam

Due to their random distribution of pores, foams have a complex internal structure and indeed thin walls and a large empty volume of up to 90% could be required. Consequently, building via AM techniques is challenging and there is a high probability of faulty parts. Therefore, a proper strategy at the design stage should be adopted. For this purpose, three rules were proposed when LPBF is used:

- Interconnection of inner pores is mandatory.
- A minimum solid fraction preventing the collapse of the structure must be offered at any layer.
- The wall thickness must be larger with respect to the effective melting diameter.

**Figure 1:**
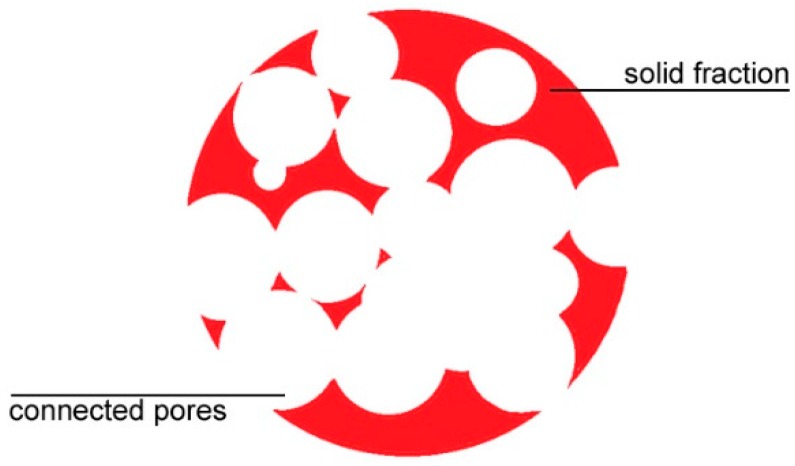
Cross-section of a sample with reduced solid fraction due to connected pores.

The first rule is a technical requirement for the extraction of the residual metal powder. The second and third rules are conceived to allow effective, rather than ineffective, manufacturing of the parts. For example, a sensible balancing between solid fraction and connected pores (Figure 1) is required to prevent collapse of the structure under its own weight where inner supporting structures must be avoided. Moreover, a minimum size of bulky material must be allowed between adjacent pores to offer local strength to effectively support the next building layers.

With respect to the third rule, the effective melting diameter of the laser beam must be evaluated in advance via preliminary quality job [6] in order to assess the actual resolution of the process, walls thinner than the actual size of a single scanning line are not possible. Further investigation resulting in additional precautions and guidelines for design are reported in the relevant section of this article.

#### 2.1.1. Generating the Solid Model

**Figure 2:**
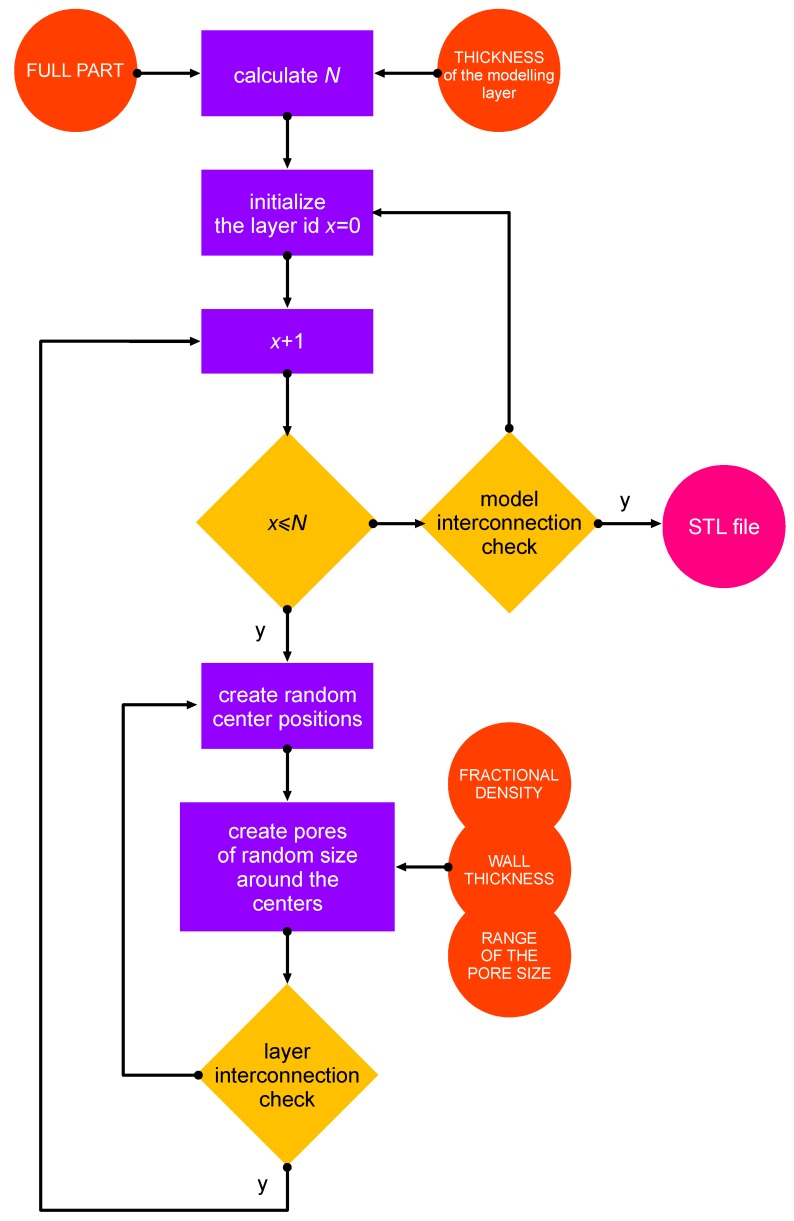
Flowchart of the algorithm to design a random foam structure.

**Figure 3:**
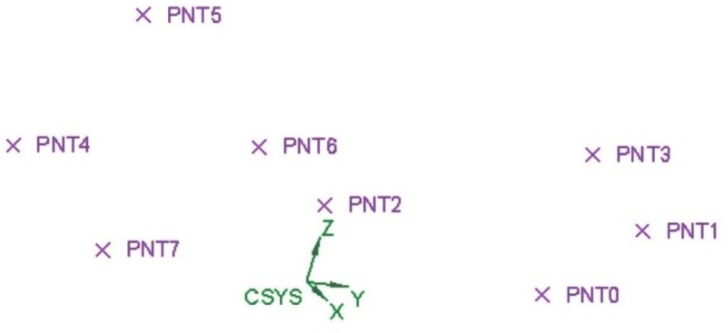
Generation of random points for pore centers, isometric view.

**Figure 4:**
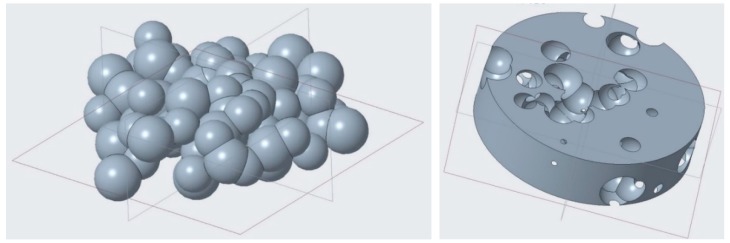
Generation of spherical pores for the current modelling layer, with resulting porous structure.

At first, to design a random foam, a flowchart was proposed (Figure 2). The algorithm was fed with crucial input data, i.e., a range for the pore size, and the aimed fractional density of the foam and the wall thickness depending on the accuracy and the resolution of the printing machine. The driving idea was to build the CAD model by means of piling up N modelling layers, which were required to comply with the referred building rules and the aimed fractional density. The number of modelling layers was based on the height of the structure being designed, as well as the available computing power. Calculations were performed using a developed macro software (Excel, 2016, Microsoft, Redmond, WA, USA), where the range of the pore diameter was given and a cloud of random pore centers was generated (Figure 3) in a cylindrical coordinate system within each the layers, from layer 1 to N. Spherical pores of random size were provided (see Figure 4), in compliance with both the constraints of fractional density and the wall thickness. If the condition of interconnection was matched, the next modelling layer was generated, otherwise, the current layer was deleted and regenerated. The process was repeated up to the actual size of the sample. Then, a final check on the interconnection was conducted. Eventually, the solid CAD model was generated.

#### 2.1.2. Generating the STL File

**Figure 5:**
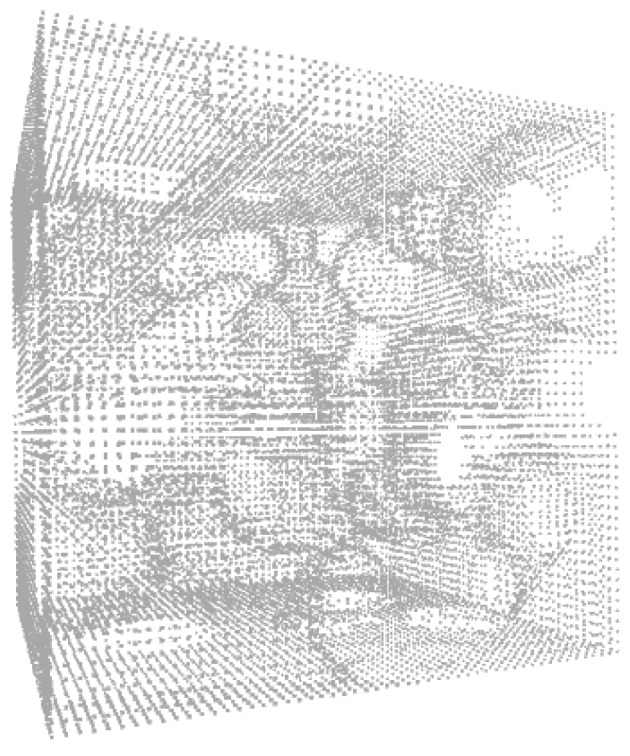
Point cloud for a cubic random foam structure.

**Figure 6:**
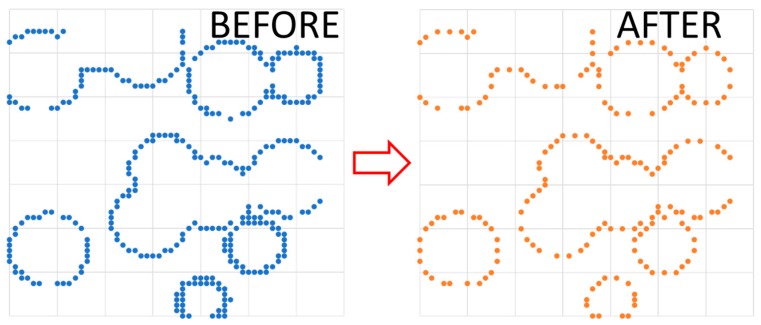
Processing a cloud of points using the approach of Pauly.

A number of steps must be addressed to effectively build the part via AM, irrespective of the manufacturing technology. Indeed, a proper mesh was required and was provided to the printing machine via an STL (standard triangle language) file [20]. Therefore, at first the solid model was converted in a point cloud to be meshed at next step, points located on the outside skin of the solid CAD model and strategically gathered along high-curvature surfaces. Nevertheless, when shifting this method to a random foam structure (Figure 5), many points were generated and most of them were redundant and ineffective to the overall precision. Therefore, the general method was deemed to be time consuming and the probability of generating defects in the mesh and specific errors of inverted normal vectors or bad edges was expected to be high. With a goal of optimized cloud of points, the approach of Pauly [21] was referred to and implemented. Using this method which considers incremental and hierarchical clustering, iterative simplification, and particle simulation algorithms to create approximations of point-based models, the number of points significantly decreased (Figure 6), the geometry was not affected and the occurrence of noise points was reduced.

The second main step was meshing the cloud of points. The constrained Delaunay triangulations (CDT) [14] was used as a reference and it was conveniently adjusted to meet the requirements of building using a foam structure, where distortion of triangles may result around the pores. As expected, a mismatch was found between the theoretical original spherical surface in the solid model and its approximation upon triangularization, i.e., a chordal error resulted on each pore and had to be reduced to improve the quality of the mesh. To this purpose, new vertices needed to be created when the chordal error exceeded a certain threshold. However, the element size used to achieve a given limit chordal error may have been very small, and therefore refining the mesh would have resulted in increased unmanageable geometrical data. One may assume the chordal error was accepted when the order of magnitude of the accuracy of the printing process was matched.

Additionally, general errors such as inverted outpointing normal vectors and poor connections of edges had to be addressed, which involved regenerating the triangle and stitching the vertexes, respectively. In general, for foam structures there was no need for specific guidance in addition to the common rules for smoothing [22]. The STL file was then sliced before processing.

#### 2.1.3. Slicing the Model

Once the building direction had been set for the building layers, the solid 3D part was converted to 2D slices. The total number of slices depended on the overall height of the structure and the thickness of the building layer, which was different from the thickness of the modelling layer. In LPBF, the latter was a compromise between the penetration depth of the laser beam and the mean particle size of the metal powder to lay [23].

**Figure 7:**
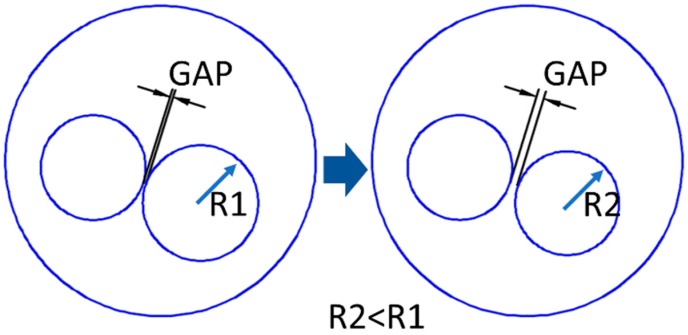
Gap between adjacent pores, before and after correction of the wall thickness.

Thin lines in each slice could result in manufacturing defects when the laser beam was scanned along it, and therefore additional actions were taken for the purpose of manufacturability. For each layer, the wall thickness, i.e., the gap between adjacent pores, was considered and compared to the actual resolution of the building process, which depended on the effective melting diameter. When the threshold had not been matched, the radius of the adjacent pores was reduced (Figure 7) to allow effective building of a solid gap. Upon correction, any change to the fractional density was negligible.

### 2.2. Manufacturing of the Foams

An EOSINT M270 laser sintering system (EOS, Krailling, Germany) with Yb-fibre laser source was used to manufacture the test article. A prealloyed, argon-atomized virgin commercial EOS GP1 stainless steel powder, 36 μm mean grain size, corresponding to standard UNS S17400 chromium copper precipitation hardening steel in terms of nominal chemical composition was used [6]. High strength, good corrosion resistance, good mechanical properties at temperatures up to 316 °C, and good toughness were offered. Indeed, this material is generally used in chemical and petrochemical industry, as well as in aerospace and marine, food processing and power plants [24].

Processing parameters (Table 1) and scanning strategies were based on preliminary trials aimed to optimize the process for the purpose of a full dense structure. An accuracy of 0.02 mm and a minimum size of building diameter of 0.190 mm were checked and these were used to address the issues of chordal error and wall thickness, respectively, although the mechanical stability during building is highly dependent on geometry and must be discussed on a case-by-case basis.

To prevent oxidation during the process, a controlled nitrogen atmosphere was arranged, the oxygen content being taken below 0.8%.

## 3. Results and discussion

### 3.1. Modelling of a Cylindrical Random Pore Foam

To test the algorithm and find any possible strategy to fix the procedure, a metal cylindrical random pore foam has been considered. A nominal diameter of 20 mm and height of 50 mm, for a total volume of 15700 mm^3^, have been set. Then, spherical random pores have been generated in the solid volume, the pore diameter has been conveniently set to range between 1 and 6 mm to prevent supporting in LPBF, the minimum allowed diameter being 8 mm. The centers of the pores have been generated in a cylindrical coordinate system aiming to a fractional density of 40 ± 2%.

**Figure 8:**
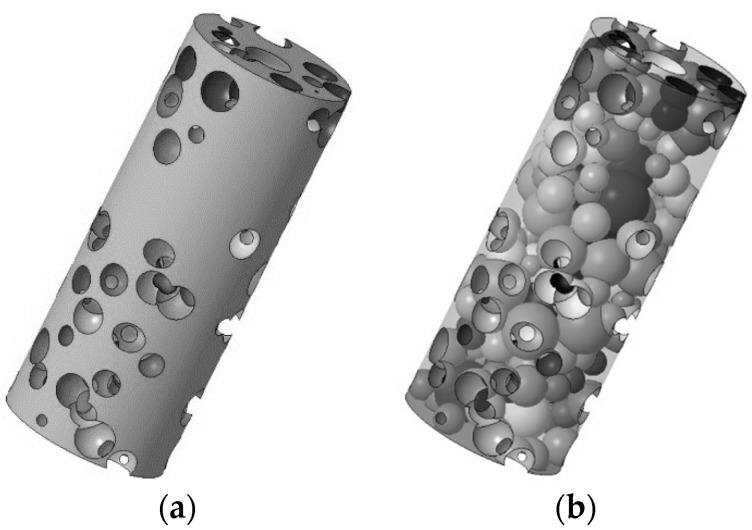
Random pore foam: (**a**) solid fraction, (**b**) distribution of pores.

For the purpose of generating the solid model, 20 modelling layers has been chosen, each one being 2.5 mm thick. Three complete iteration cycles of the algorithm have been required to generate the structure to be built. It is worth noting that several pores intersecting the outer skin are required in order to allow powder ejection during building, moreover, as required, interconnection among the pores is mandatory (Figure 8).

**Figure 9:**
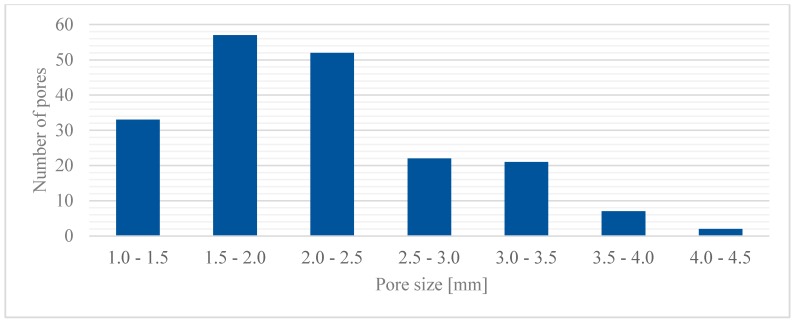
Pore size distribution in the model of the random foam.

A total of 192 pores has been generated (Figure 9) and the highest frequencies of occurrence have been found for the groups with pore size between 1.5 and 2.5 mm. On the other hand, the lowest frequency resulted for the groups with pores ranging between 3.5 and 6.0 mm. Indeed, larger pores are unfavorable as they would result in reduced local strength, and therefore they would not comply with the basic rules of the algorithm.

**Figure 10:**
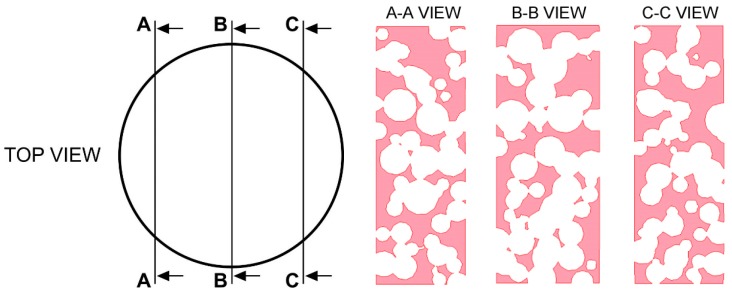
Virtual longitudinal sections at 25% (A-A view), 50% (B-B view) and 75% (C-C view) volume cut.

A bulky 6579.06 mm^3^ volume resulted, thus yielding a fractional density of 41.88%. Interconnection of the pores can be checked by means of virtual longitudinal sections (Figure 10) at 25%, 50%, and 75% volume cut.

**Figure 11:**
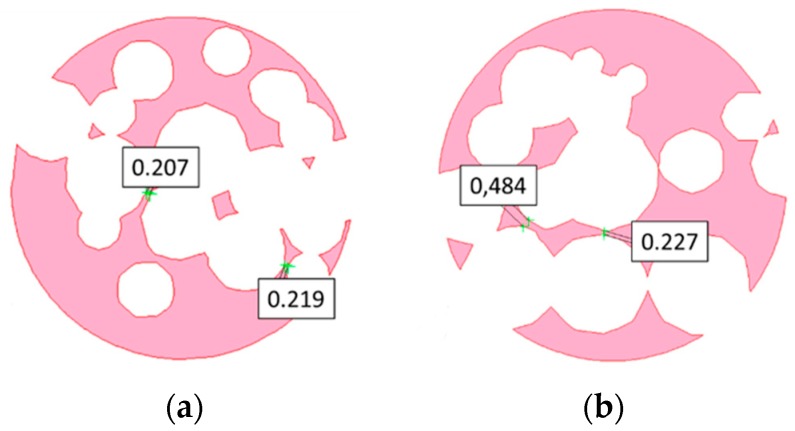
Virtual transverse cross-sections at (**a**) 10 mm and (**b**) 25 mm height.

Further checks must be conducted on transverse cross-sections, as an example, virtual cuts at 10 mm and 25 mm height are considered (Figure 11) when: the solid fraction is effective to support the next layers, the wall size exceeds a minimum threshold of 190 µm, and the maximum chordal error is 0.15 mm.

### 3.2. Building of a Cylindrical Random Pore Foam

**Figure 12:**
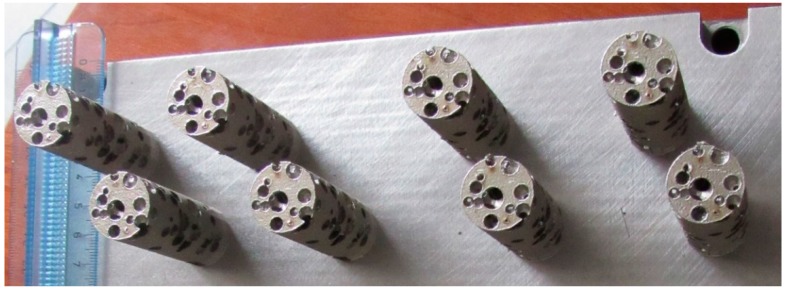
Cylindrical random foams manufactured by means of LPBF.

**Figure 13:**
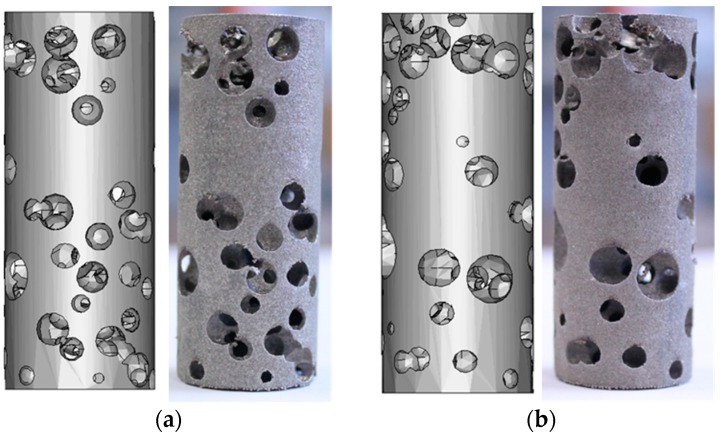
Comparing the nominal solid model to the built foam, (**a**) and (**b**) are views of opposite sides.

The optimized STL source file has been used to manufacture eight samples (Figure 12) by means of LPBF, for which a natural direction of growth has been considered with the axis of the cylinders being orthogonal to the building plate, thus preventing supporting structures. The nominal model has been compared with the built foam (Figure 13), in terms of pore size and wall thickness, aiming to check the reliability of the building process and possibly update the design algorithm.

**Figure 14:**
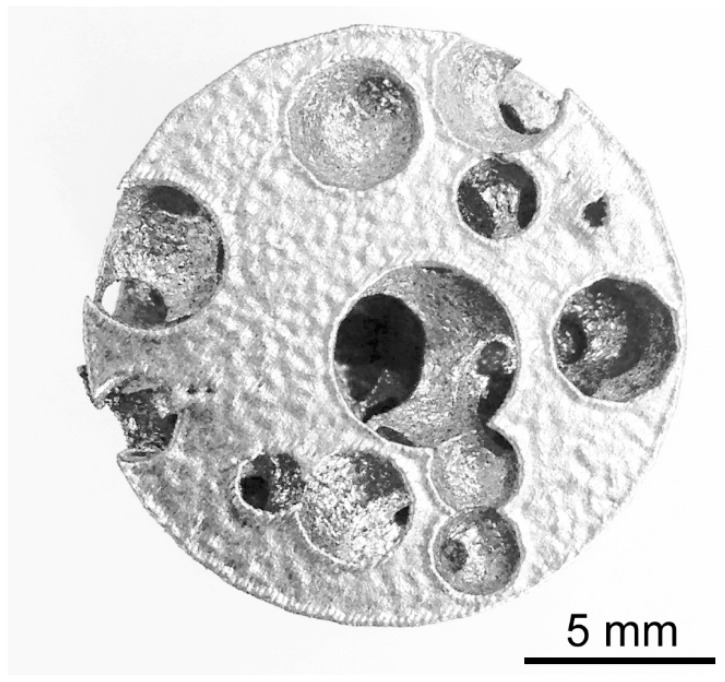
Example of a cross-cut section of the sample foam.

**Figure 15:**
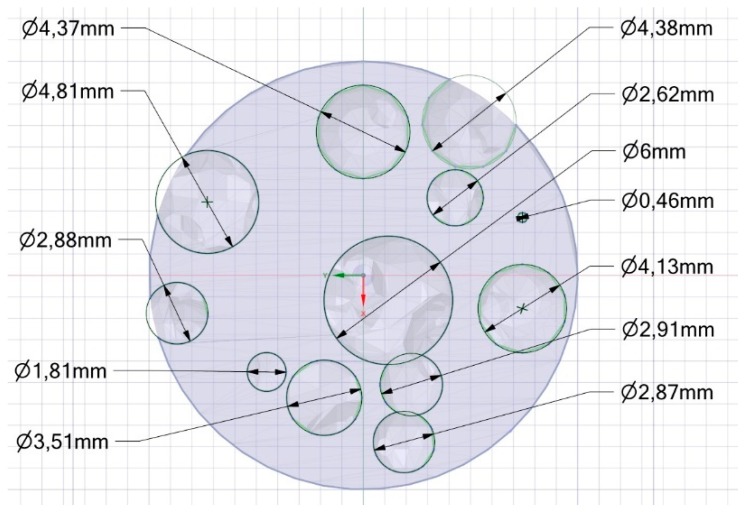
Example of the nominal cross-section corresponding to the actual cross-cut section.

Transverse cross-cuts of the samples have been made at a given height (Figure 14). Since the direction of building is parallel to the longitudinal axis of a sample, these cuts are made in a plane which is parallel to the building layer. The resulting circular cross-sections have been compared to their counterpart in the nominal model at the same height (Figure 15). To be specific, the average diameter of each circle (i.e., each section of a pore) has been measured by optical microscopy (Table 2) and the percentage absolute mismatch has been evaluated. It is worth noting that although a range is set for the pore size in the design algorithm, diameters below the lower limit may be found when the cut is close to the pole of the spherical pore. 

**Figure 16:**
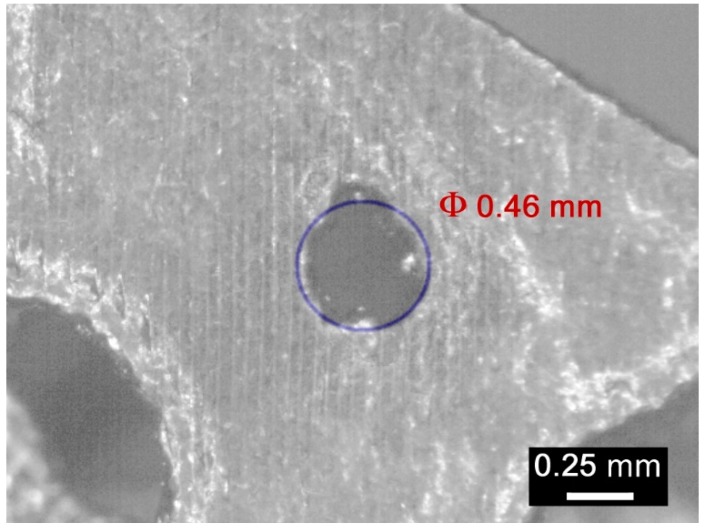
Detail of roundness error for the circle of 0.46 mm nominal diameter.

**Figure 17:**
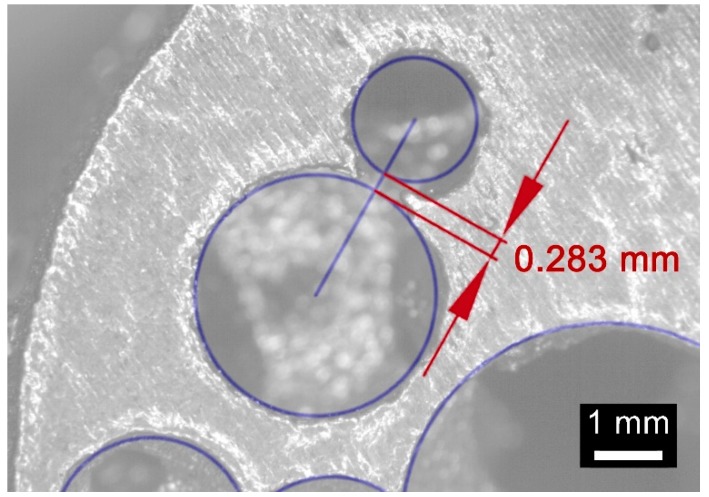
Detail of collapse at the interface between pores, nominal geometry is superimposed.

An average absolute mismatch of 4.0% resulted, and two reasons can be inferred for conditions of mismatch above 5%. At first, the resolution of the printing machine is a factor in the roundness error when small circles, (i.e., at the poles of a sphere, for a circle of 0.46 mm nominal diameter) must be drawn (Figure 16). Moreover, high percentage mismatch could result as a consequence of wall collapse, in fact, a region of major defect was found between the interfaces of pores with nominal diameters of 1.81 and 3.51 mm (Figure 17), where wall thickness of 0.283 mm was set in the model. On the basis of this, it may be assumed that the constraint given to wall thickness in the design algorithm of a metal foam, must be shifted from 0.190 mm to 0.300 mm at least.

For the final purpose of checking the fraction density, weighing has been performed and the density has been measured via the Archimede method. An average weight of 52.786 g resulted; and given a reference full density of 7.9 g/cm^3^, an average volume of 6681 mm^3^ resulted, thus yielding to a 1.5% error with respect to the nominal model. The mismatch is thought to be affected by a small quantity of trapped powder inside the specimen and a minor geometric internal error.

## 4. Conclusions

In this paper, an approach to design and build random foam structures with interconnected porosity has been presented. The strategy has been optimized for additive manufacturing via laser powder bed fusion. For this purpose, several rules have been proposed. Namely, at the design stage, a general algorithm has been developed and tested to model a random foam structure using technical and manufacturing constraints such as the range of the pore size, the wall thickness, and the aimed fractional density. The latter depends on the specific application of the foam.

In this phase, a combination of constrained Delaunay triangulations and the approach of Pauly has been implemented to reduce the number of modelling points, and therefore the total size of the STL file has benefited.

To test the approach, a steel cylindrical random foam has been designed and built. Good agreement with the nominal model source file has been achieved, with minor errors of approximately 4.0%, on average, for circle diameter. Crucial findings have been drawn to update the algorithm for model generation, nevertheless, the intended volume, hence the intended density, has been matched in this research with an overall 1.5% mismatch. This approach of designing random pore distributions within a given bulk volume can be used to model any complex structure where inner interconnected porosity is required in the form of random foam for the purpose of lightening the structure. A check of manufacturability is the preliminary step before developing a structured experimental plan to further investigate the impact of pore distribution on the mechanical properties.

## Figures and Tables

**Table 1:** Processing parameters in laser powder bed fusion (LPBF) of EOS GP1 stainless steel powder.

Factor	Value
Laser power	195 W
Scanning speed	0.75 m/s
Hatch spacing	100 μm
Scan length	20 mm
Layer thickness	20 μm

**Table 2:** Nominal vs average actual circle diameter (ordered by size) and corresponding mismatches.

Nominal (mm)	Actual (mm)	Mismatch (%)
0.46	0.51	8.9
1.81	1.97	8.8
2.62	2.71	3.2
2.87	2.91	1.4
2.88	2.98	3.6
2.91	3.07	5.3
3.51	3.79	8.1
4.13	4.12	0.0
4.37	4.45	1.8
4.38	4.32	1.4
4.81	4.92	2.2
6.00	6.17	2.8
